# Scaling Graph Propagation Kernels for Predictive Learning

**DOI:** 10.3389/fdata.2022.616617

**Published:** 2022-04-08

**Authors:** Priyesh Vijayan, Yash Chandak, Mitesh M. Khapra, Srinivasan Parthasarathy, Balaraman Ravindran

**Affiliations:** ^1^School of Computer Science, McGill University, Montreal, QC, Canada; ^2^Mila—Quebec AI Institute, Montreal, QC, Canada; ^3^College of Information and Computer Sciences, University of Massachusetts Amherst, Amherst, MA, United States; ^4^Department of Computer Science and Engineering, Robert Bosch Centre for Data Science, Indian Institute of Technology Madras, Chennai, India; ^5^Department of Computer Science and Engineering, Department of Biomedical Informatics, Ohio State University, Columbus, OH, United States

**Keywords:** graph neural network, semi-supervised learning (SSL), node classification, social network analysis, deep learning—artificial neural network (DL-ANN)

## Abstract

Many real-world applications deal with data that have an underlying graph structure associated with it. To perform downstream analysis on such data, it is crucial to capture relational information of nodes over their expanded neighborhood efficiently. Herein, we focus on the problem of Collective Classification (CC) for assigning labels to unlabeled nodes. Most deep learning models for CC heavily rely on differentiable variants of Weisfeiler-Lehman (WL) kernels. However, due to current computing architectures' limitations, WL kernels and their differentiable variants are limited in their ability to capture useful relational information only over a small expanded neighborhood of a node. To address this concern, we propose the framework, I-HOP, that couples differentiable kernels with an iterative inference mechanism to scale to larger neighborhoods. I-HOP scales differentiable graph kernels to capture and summarize information from a larger neighborhood in each iteration by leveraging a historical neighborhood summary obtained in the previous iteration. This recursive nature of I-HOP provides an exponential reduction in time and space complexity over straightforward differentiable graph kernels. Additionally, we point out a limitation of WL kernels where the node's original information is decayed exponentially with an increase in neighborhood size and provide a solution to address it. Finally, extensive evaluation across 11 datasets showcases the improved results and robustness of our proposed iterative framework, I-HOP.

## 1. Introduction

Many real-world datasets such as social networks can be modeled using a graph wherein the nodes in the graph represent entities in the network and edges between the nodes capture the interactions between the corresponding entities. Furthermore, every node can have attributes associated with it and some nodes can have known labels associated with them. Given such a graph, collective classification (CC) (Neville and Jensen, 2000; Lu and Getoor, 2003; Sen P et al., 2008) is the task of assigning labels to the remaining unlabeled nodes in the graph. A key task here is to extract relational features for every node which not only consider the attributes of the node but also the attributes and labels of its partially labeled neighborhood. Neural network based models have become popular for computing such node representations by aggregating node and neighborhood information.

The key idea is to exploit the inherent relational structure among the nodes which encodes valuable information about homophily, influence, community structure, etc. (Jensen et al., 2004). Traditionally, various neighborhood statistics on structural properties (Gallagher and Eliassi-Rad, 2008), and distributions on labels and features were used as relational features to predict labels (Lu and Getoor, 2003; Neville and Jensen, 2003; McDowell and Aha, 2013). Furthermore, iterative inference techniques were widely adopted to propagate these label predictions until convergence (Sen P et al., 2008). Recently, Kipf and Welling (2017) proposed Graph Convolutional Networks (GCN) with a Laplacian based graph kernel for the node classification task. GS (Hamilton et al., 2017) further extended GCN and proposed few additional neighborhood aggregation functions to achieve state-of-the-art results for inductive learning.

These graph convolution kernels are based on differentiable extensions of the popular Weisfieler-Lehman(WL) kernels. In this study, we first show that a *direct adaptation of WL kernels for the CC task is inherently limited as node features get exponentially morphed with neighborhood information* when considering farther hops. More importantly, aggregating information from a *K*-hop neighborhood in an end-to-end differentiable manner is not easily scalable. The exponential increase in neighborhood size with an increase in hops severely limits the model due to excessive memory and computation requirements. To address these, we propose an Iterative Higher-order Propagation framework (I-HOP) that provides a solution for both these problems. Our main contributions are:

- *A modular graph kernel* that generalizes many existing methods. Through this, we discuss a *Node Information Morphing (NIM)* phenomenon. We discuss its implications on the limitations of existing methods and then discuss a novel family of kernels called the *Node Information Preserving (NIP) kernels* to address these limitations.
- A *hybrid semi-supervised learning framework* (I-HOP) for higher order propagation that couples differentiable kernels with an iterative inference procedure to aggregate neighborhood information over farther hops. This allows differentiable kernels to *exploit label information* and further *overcome excessive memory constraints* imposed by multi-hop information aggregation.
- An extensive *experimental study on 11 datasets* from different domains. We demonstrate the node information morphing issue and show that *the proposed Iterative NIP model is robust* and overall outperforms existing models.

## 2. Background

In this section, (i) we define the notations and terminologies used, (ii) we present the generic differentiable kernel for capturing higher order information in the CC setting, (iii) we discuss existing works in the light of the generic kernel, and (iv) analyze the NIM issue.

### 2.1. Definitions and Notations

Let *G* = (*V, E*) denote a graph with a set of vertices, *V*, and edges, *E* ⊆ *V* × *V*. Let |*V*| = *n*. The set E is represented by an adjacency matrix *A* ∈ ℝ^*n* × *n*^, and let *D* ∈ ℝ^*n* × *n*^ denote the diagonal degree matrix defined as $D_{ii}=\underset{j}{\sum }A_{i,j}$.

A CC dataset defined on graph *G* comprises of a set of labeled nodes, *S*, a set of unlabeled nodes, *U* with *U* = *V* − *S*, a feature matrix: *X* ∈ ℝ^*n* × *f*^ and a label matrix: *Y* ∈ {0, 1}^|*S*| × *l*^, where *f* and *l* denote the number of features and labels, respectively. Let Ŷ ∈ ℝ^*n* × *l*^ denote the predicted label matrix.

In this study, neural networks defined over *K*-hop neighborhoods have *K* aggregation or convolution layers with *d* dimensions each and whose outputs are denoted by *h*_1_, …, *h*_*K*_. We denote the learnable weights associated with *k*th layer as $W_{k}^{\phi }$ and $W_{k}^{\psi }\in {\mathbb{R}}^{d\times d}$. The weights of the input layer ($W_{1}^{\phi }$, $W_{1}^{\psi }$) and output layer, *W*_*L*_ are in ℝ^*f* × *d*^ and ℝ^*d* × *l*^, respectively. Iterative inference steps are indexed by *t* ∈ (1, *T*).

### 2.2. Generic Propagation Kernel

We define the generic propagation (graph) kernel as follows:


(1) $$\begin{array}{l} \begin{array}{l} h_{0}=X\\ h_{k}=\sigma_{k}\left(\alpha \cdot \left(\Phi_{k}\cdot W_{k}^{\phi }\right)+\beta \cdot \left(F\left(A\right)\cdot {\text{Ψ}}_{k}\cdot W_{k}^{\psi }\right)\right) \end{array} \end{array}$$

where Φ_*k*_ and Ψ_*k*_ are the node and neighbor features considered at the *k*th propagation step (layer), *F*(*A*) is a function of the adjacency matrix of the graph, and $W_{k}^{\phi }$ and $W_{k}^{\psi }$ are weights associated with the *k*th layer of the neural network. One can view the first term in the equation as processing the information of a given node and the second term as processing the neighbors' information. The kernel recursively computes the outputs of the *k*th layer by combining the *features* computed to the (*k* − 1)th layer. σ_*k*_ is the activation function of the *k*th, layer and α and β can be scalars, vectors, or matrices depending on the kernel.

Label predictions, Ŷ can be obtained by projecting *h*_*K*_ onto the label space followed by a sigmoid or softmax layer corresponding to a multi-class or multi-label classification task. The weights of the model are learned *via* backpropagation by minimizing an appropriate classification loss on Ŷ.

### 2.3. Relation to Existing Works

Appropriate choice of α, β, Φ, Ψ, and *F*(*A*) in the generic kernel yield different models. Table 1 lists out the choices for some of the popular models and our proposed approaches. Iterative collective inference techniques, such as the Iterative Classification Algorithm (ICA) family combine node information with aggregated label summaries of immediate neighbors to make predictions. Aggregation can be based on averaging kernel: *F*(*A*) = *D*^−1^*A*, or label count kernel: *F*(*A*) = *A*, etc. with labels as neighbors' features (Ψ_*k*_ = Ŷ). This neighborhood information is then propagated iteratively to capture higher order information. ICA also has a semi-supervised variant (McDowell and Aha, 2012) where after each iteration the model is re-learned with updated labels of neighbors. Table 1 shows how the modular components can be chosen to see semi-supervised ICA (SS-ICA) as a special instantiation of our framework.

**Table 1:** Baselines, existing, and proposed models seen as instantiations of the proposed framework.

**Models**	**Φ_*k*_**	**F(A)**	**Ψ_*k*_**	**α**	**β**	** $W_{k}^{\phi }=W_{k}^{\psi }?$ **	**Differentiable Kernel**	**Iterative Inference**
BL_NODE	*h*_0_	-	-	1	-	-	-	No
BL_NEIGH	-	*D* ^−1^ *A*	*h*_*k*−1_	-	1	-	Yes	No
SS-ICA	*h*_0_	*D* ^−1^ *A*	Ŷ	1	1	No	No	Yes
WL	*h*_*k*−1_	*A*	*h*_*k*−1_	1	1	-	-	No
GCN	*h*_*k*−1_	(*D* + *I*)^−1/2^*A*(*D* + *I*)^−1/2^	*h*_*k*−1_	(*D* + *I*)^−1^	1	Yes	Yes	No
GCN-MEAN	*h*_*k*−1_	*D* ^−1^ *A*	*h*_*k*−1_	1	1	Yes	Yes	No
GS-Pool	*h*_*k*−1_	*maxpool*	*h*_*k*−1_	1	1	No	Yes	No
GS-MEAN	*h*_*k*−1_	*D* ^−1^ *A*	*h*_*k*−1_	1	1	No	Yes	No
GS-LSTM	*h*_*k*−1_	LSTM gates	LSTM	1	1	No	Yes	No
NIP-MEAN	*h*_0_	*D* ^−1^ *A*	*h*_*k*−1_	1	1	No	Yes	No
I-HOP-MEAN	*h*_0_	*D* ^−1^ *A*	*h*_*k*−1_, Ŷ	1	1	No	Yes	Yes

The WL family of recursive kernels (Weisfeiler and Lehman, 1968; Shervashidze et al., 2011) were initially defined for graph isomorphism tests and most recent CC methods use differentiable extensions of it. In its basic form, it is the simplest instantiation of our generic propagation kernel with no learnable parameters as shown in Table 1.

The modified, normalized symmetric Laplacian kernel (GCN) used in Kipf and Welling (2017) can be seen as an instance of the generic kernel with node weight, α = (*D* + *I*)^−1^, individual neighbors' weights' *F*(*A*) = (*D* + *I*)^−1/2^*A*(*D* + *I*)^−1/2^, Φ_*k*_ = Ψ_*k*_ and $W_{k}^{\phi }=W_{k}^{\psi }$. We also consider its mean aggregation variant (GCN-MEAN), where *F*(*A*) = *D*^−1^*A*. In theory, by stacking multiple graph convolutional layers, any higher order information can be captured in a differentiable way in *O*(*K* × *E*) computations. However, in practice, the proposed model by Kipf and Welling (2017) is only full batch trainable and, thus, cannot scale to the large graph when memory is limited.

Hamilton et al. (2017) proposed GraphSAGE (GS) different variants of *k*th order differentiable WL kernels, viz: GS-MEAN, GS-Pool, and GS-LSTM. These variants can be viewed as special instances of our generic framework as mentioned in Table 1. GS-Pool applies a max-pooling function to aggregate neighborhood information whereas GS-LSTM uses an LSTM to combine neighbors' information sequenced in random order similar to the study by Moore and Neville (2017). GS has a mean averaging variant, similar to the GCN-MEAN model but treats nodes separately from its neighbors, i.e., $W_{k}^{\phi }\neq W_{k}^{\psi }$. Finally, it either concatenates or adds up the node and neighborhood information. GS-LSTM is over-parameterized for small datasets. With GS-MAX and GS-LSTM there is a loss of information as Max pooling considers only the largest input and LSTM focuses more on the recent neighbors in the random sequence.

We also provide an extended study of related works in section: B of the supplementary material that discusses other graph kernels, message passing models, and similar analysis.

## 3. Node Information Morphing

In this section, we show that existing models which extract relational features, *h*_*k*_ do not retain the original node information, *h*_0_ completely. With multiple propagation steps, the *h*_0_ is decayed and morphed with neighborhood information. We term this issue as NIM.

For ease of illustration, we demonstrate the NIM issue by ignoring the non-linearity and weights. Based on the commonly observed instantiations of our generic propagation kernel [Equation (1)], where Φ_*k*_ = Ψ_*k*_ = *h*_*k*−1_, we consider the following equation:


(2) $$\begin{array}{l} h_{k}=\alpha *Ih_{k-1}+\beta *F\left(A\right)h_{k-1} \end{array}$$

On unrolling the above expression, one can derive the following binomial form:


$$\begin{array}{l} h_{k}=\left(\alpha *I+\beta *F\left(A\right)\right)h_{k-1} \end{array}$$

(3) $$\begin{array}{l} h_{k}={\left(\alpha *I+\beta *F\left(A\right)\right)}^{k}h_{0} \end{array}$$

From Equation (3), it can be seen that the relative importance of information associated with the node's 0th hop information, *h*_0_, is $\frac{\alpha^{k}}{{\left(\alpha +\beta \right)}^{k}}$. Hence, for any positive β, the importance of *h*_0_ decays exponentially with *k*. It can be seen that the decay rate for GCN is (*D* + *I*)^−*k*^ and (2)^−*k*^ for the other WL kernel variants mentioned in Table 1.

### 3.1. Skip Connections and NIM

It can be similarly derived and seen that the information morphing not only happens at *h*_0_ but also for every *h*_*k*_∀*k* ∈ [0, *K* − 1]. This decay of neighborhood information can be reduced by leveraging skip connections. Consider the propagation kernel in Equation (2) with skip connections as shown below:


(4) $$\begin{array}{l} h_{k}=\left(\alpha *Ih_{k-1}+\beta *F\left(A\right)h_{k-1}\right)+h_{k-1} \end{array}$$

The above equation on expanding as above gives:


(5) $$\begin{array}{l} h_{k}={\left(\left(\alpha +1\right)*I+\beta *F\left(A\right)\right)}^{k}h_{0} \end{array}$$

The relative importance of weights of *h*_0_ then becomes $\frac{{\left(\alpha +1\right)}^{k}}{{\left(\alpha +\beta +1\right)}^{k}}$, which decays slower than $\frac{\alpha^{k}}{{\left(\alpha +\beta \right)}^{k}}$ for all α, β > 0. Though this helps in retaining information longer, it does not solve the problem completely. Skip connections were also used in GCN to reduce the drop in performance of their model with multiple hops. The addition of skip connection in GCN was originally motivated from the conventional perspective to avoid the reduction in performance with increasing neural network layers and not with the intention to address information morphing. In fact, their standard 2 layer model cannot accommodate skip connections because of varying output dimensions of layers. Similarly, GS models which utilized concatenation operation to combine node and neighborhood information also lessened the decay effect in comparison to summation based combination models. This is because concatenation of information from the previous layer can be perceived as skip connections, as noted by its authors. Though the above analysis is done on a linear propagation model, this insight is applicable to the non-linear models as well. Our empirical results also confirm this.

### NIP Models

To address the NIM issue, we propose a specific class of instantiations of the generic kernel which we call the NIP models. One way to avoid the NIM issue is to explicitly retain the *h*_0_ information at every propagation step as in Equation (6). This is obtained from Equation (1) by setting Φ_*k*_ = *h*_0_ and Ψ_*k*_ = *h*_*k*−1_, ∀*k*. For different choices of α, β, and *F*(*A*), we get different kernels of this family. In particular, setting β = 1 − α and *F*(*A*) = *D*^−1^*A* yields a kernel similar to Random Walk with Restart (RWR) (Tong et al., 2006), refer to Equation (7).


(6) $$\begin{array}{l} h_{k}=\alpha h_{0}W_{k}^{\phi }+\beta F\left(A\right)h_{k-1}W_{k}^{\psi } \end{array}$$

(7) $$\begin{array}{l} h_{k}=\alpha h_{0}+\beta F\left(A\right)h_{k-1} \end{array}$$

The NIP formulation has two significant advantages: (a) It enables capturing correlation between *k*-hop reachable neighbors and the node explicitly and (b) it creates a direct gradient path to the node information from every layer, thus allowing for better training. We propose a specific instantiation of the generic NIP kernel below.


(8) $$\begin{array}{l} \text{NIP-MEAN}:h_{k}=\sigma \left(h_{0}W_{k}^{\phi }+D^{-1}Ah_{k-1}W_{k}^{\psi }\right) \end{array}$$

Node Information Preserving-MEAN is similar to GCN-MEAN but with Φ_*k*_ = *h*_0_ and $W_{k}^{\phi }\neq W_{k}^{\psi }$.

## 4. Iterative Higher-Order Propagation

Building any end-to-end differentiable model requires all the relational information to be in memory. This hinders models with a large number of parameters and those that process data in large batches. For graphs with high link density and a power law degree distribution, processing even 2nd or 3rd hop information becomes infeasible. Even with *p*-regular graphs, the memory grows at *O*(*p*^*K*^) with the number of hops, *K*. Thus, using a differentiable kernel for even a small number of hops over a moderate size graph becomes infeasible.

To address this critical scalability issue, we propose a novel I-HOP Framework, which encompasses the differentiable kernels within an iterative mechanism. In each iteration of I-HOP, the differentiable kernel computes a *C* hop neighborhood summary, where *C* < *K*. Every iteration starts with a summary, Θ^*t*−1^, of the information computed until the (*t* − 1) step as given below.


(9) $$\begin{array}{l} h_{0}^{0}=X;\Theta^{0}=0\\ h_{k}^{t}=\sigma \left(\alpha *\Phi_{k}W_{k}^{\phi }+\beta *F\left(A\right){\text{Ψ}}_{k}^{t}W_{k}^{\psi }\right)\\ {\text{Ψ}}_{k}^{t}=\left[{\text{Ψ}}_{k},\Theta^{t-1}\right] \end{array}$$

After *T* iterations, the model would have incorporated (*K* = *T* × *C*) hop neighborhood information. Here, we fix *T* based on the required number of hops we want to capture (*K*), but it can also be based on some convergence criteria on the inferred labels. For the empirical results reported in this study, we have chosen Θ^*t*−1^ to be (predicted) labels Ŷ^*t*−1^, along the lines of the ICA family of algorithms. Other choices for Θ^*t*−1^ includes the *C* hop relational information, $h_{C}^{t}$.

**Figure 1:**
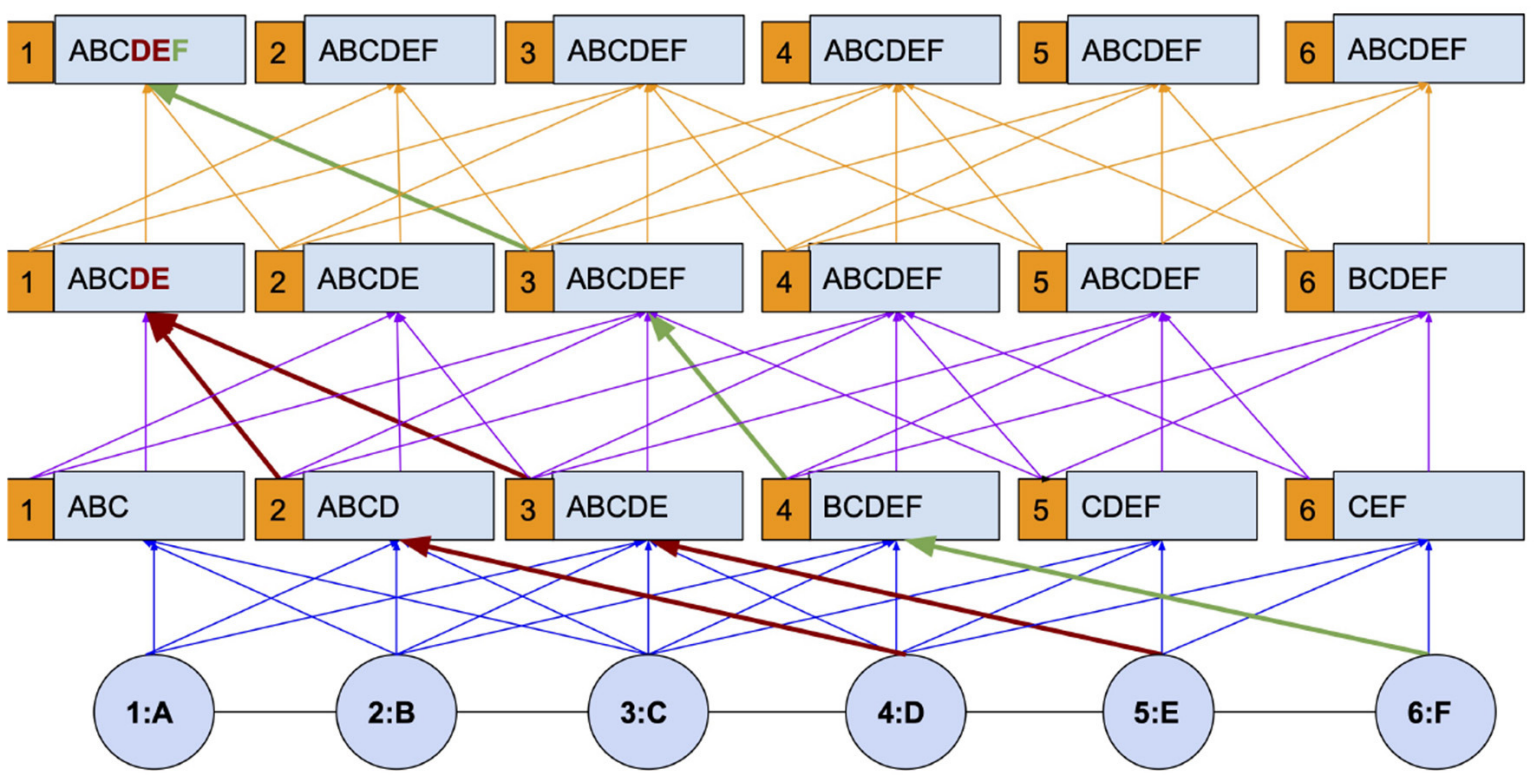
Iterative higher-order propagation (I-HOP) explained with a chain graph.

Figure 1 explains I-HOP's mechanism with a toy chain graph. The graph has 6 nodes with attributes ranging over A–F and the graph kernel used is of the second order. The figure is intended to explain how differentiable and non-differentiable layers are interleaved to allow propagation up to the diameter. We first analyze it with respect to node 1. In the first iteration, node 1 has learned to aggregate attributes from nodes 2 and 3, viz *BC*, along with its own. This provides it with an aggregate of information from A, B, and C. At the start of each subsequent iteration, label predictions are made for all the nodes using a *C*^*th*^(In Figure 1, *C* = 2) order differentiable kernel learned in the previous iteration. These labels are concatenated with node attributes to form the features for the current iteration. By treating the labels as non-differentiable entities, we stop the gradients from propagating to the previous iteration, and hence, the model is only *C* = 2 hop differentiable.

With the concatenated label information, the model can be made to re-learn from scratch or continue on top of the pre-trained model from the last iteration. Following this setup, one can observe that the information of nodes D, E, and F which is not accessible with a 2nd order differentiable kernel(blue paths) is now accessible *via* the non-differentiable paths (red and green paths). In the second iteration, information from nodes at the 3rd and 4th hop (D and E) becomes available and in the subsequent iteration, information from the 5th hop (F) becomes available. The paths encoded in blue, purple, and orange represent different iterations in the figure and are differentiable only during their ongoing iteration, not as a whole.

### 4.1. I-HOP-MEAN: Iterative NIP Mean Kernel

In this section, we propose a special instance of the I-HOP framework which addresses the NIM issue with NIP kernels in a scalable fashion. Specifically, we consider the following NIP Kernel instantiation, *I-HOP-MEAN* with mean aggregation function, by setting *F*(*A*) = *D*^−1^*A*, Φ_*k*_ = *h*_0_, Ψ = *h*_*k*−1_, Θ^*t*−1^ = Ŷ^*t*−1^ and $W_{K}^{\phi }\neq W_{K}^{\psi }$.


(10) $$\begin{array}{l} h_{0}^{0}=X;{Ŷ}^{0}=0\\ h_{k}^{t}=\sigma \left(h_{0}^{t}W_{k}^{\phi }+D^{-1}A\left[h_{k-1}^{t},{Ŷ}^{t-1}\right]W_{k}^{\psi }\right) \end{array}$$

In Algorithm 1 (I-HOP-MEAN), the iterative learning and inference steps are described in *lines: 7–10* and *12–16*, respectively. Both learning and inference happen in mini-batches, *nodes*, sampled from the labeled set, *S* or the unlabeled set, *U* respectively as shown in *lines*:8 and 12, correspondingly. The *predict* function described in *lines: 17–27* is used during learning and inference to obtain label predictions for nodes, *nodes*. The procedure first extracts *C*-hop relational features (*h*_*k*_ with *k* = *C*) and then projects it to the label space and applies a sigmoid or a softmax depending on the task (*line: 27*).

**Algorithm 1:**
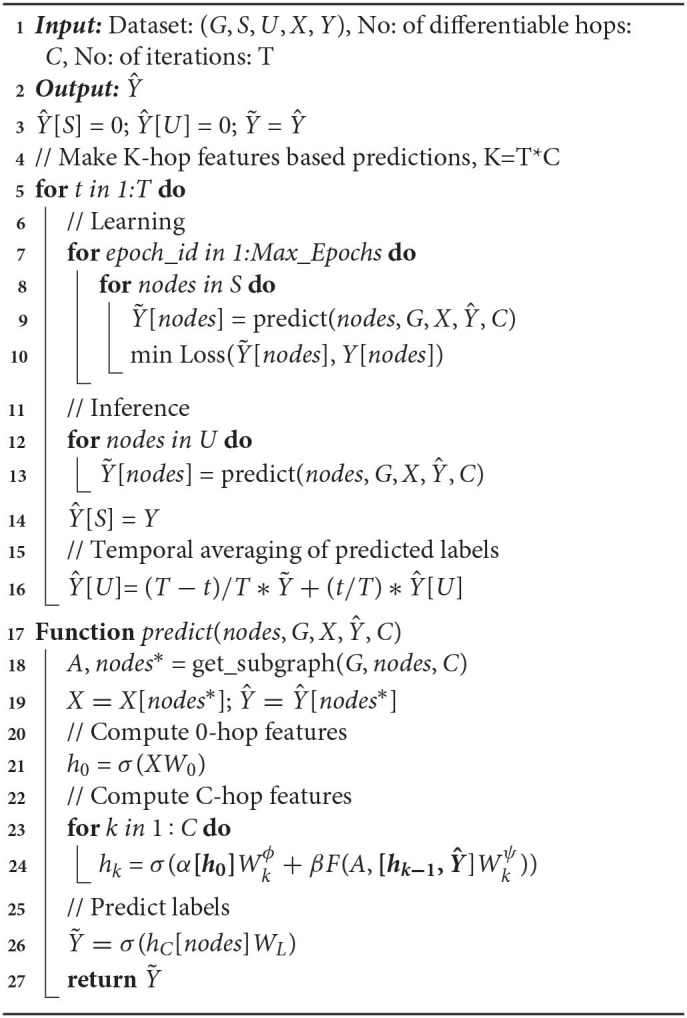
I-HOP-MEAN.

To extract *C*-hop relational features for *nodes*, the model *via* the *get_subgraph* function first gathers all *nodes* along with their neighbors reachable by less than *C* + 1 hops (nodes*) and represents this entire sub-graph by an adjacency matrix (*A*). A *C*-hop representation is then obtained with the kernel as in *lines: 21–24*. At each learning phase, the weights of the kernels ($W_{k}^{\phi }$s and $W_{k}^{\psi }$, ∀*k*) are updated *via* back-propagation to minimize an appropriate loss function.

## 5. Scalability Ananlysis

In most real-world graphs exhibiting power law, the size of the neighborhood for each node grows exponentially with the depth of the neighborhood considered. Storing all the node attributes, the edges of the graph, intermediate activations, and all the associated parameters become a critical bottleneck. Here, we analyze the efficiency of the proposed study to scale to large graphs in terms of the reduction in the number of parameters and space and time complexity.

### 5.1. Number of Parameters

The ratio of available labeled nodes to the unlabeled nodes in a graph is often very small. As observed in Kipf and Welling (2017) and Hamilton et al. (2017), the model tends to easily over-fit and perform poorly during test time when additional parameters (layers) are introduced to capture a deeper neighborhood. In our proposed framework with iterative learning and inference, the parameters of the kernel at (*t* − 1)th iteration are used to initialize the *t*-th kernel and are then discarded, hence the model parameter is *O*(*C*) and not *O*(*K*). Thus, the model can obtain information from any arbitrary hop, *K* with constant learnable parameters of *O*(*C*), where *C* = *T*/*K*. But in the inductive setup, the parameter complexity is similar to GCN and GS as the kernel parameters from all iterations are required to make predictions for unseen nodes.

### 5.2. Space and Time Complexity Analysis

For a Graph *G* = (*V, E*), let us consider aggregating information up to the *K* hop neighborhood. Let number of nodes *n* = |*V*|, and average degree *p* = 2|*E*|/*n*.

**Full-Batch GNNs:** Instantiations of the generic kernel given in Equation (1) with full batch updates over the entire graph, such as GCNs (Kipf and Welling, 2017), require *O*(2*nd*^*k*^ + 2*d*^*k*^*d*^*k*+1^) memory for each layer, *k* ∈ [1, *K*]. *O*(*nd*^*k*^) is required to save the node information, Ψ_*k*_ and the neighborhood information, Φ_*k*_ for *N* nodes in layer *k*, assuming *d*^*k*^ is the length of their features. *O*(*d*^*k*^*d*^*k*+1^) memory is required to store the weight matrices in each layer, $W_{k}^{\Phi }$ and $W_{k}^{\Psi }$. Note that, with full batch GNNs, the entire graph should also be in memory and that takes *O*(*np*) memory with sparse implementations but that is shared across all layer computations.

Full batch GNNs have a time complexity of *O*(2*nd*^*k*^*d*^*k*+1^ + *npd*^*k*+1^) at each layer, *k* where the multiplication of node and neighbor features with their corresponding weights take *O*(*nd*^*k*^*d*^*k*+1^) time complexity and the sparse-dense multiplication required to aggregate *d*^*k*+1^-dimensional neighbor features from *p* neighbors for *N* nodes take *O*(*npd*^*k*+1^) time complexity.

For our analysis, we focus only on the scalability concerning graph size. Thus, for a *K* layer GNN model with full-batch updates, the simplified space complexity and time complexity are *O*(*Kn* + *np*) and *O*(*Knp*), respectively.

**Mini-Batch GNNs:** As the number of nodes in a graph increases, the GNN's memory requirement quickly becomes impractical especially to be held in VRAM [Graphic Processing Unit (GPU) Memory]. While mini-batch implementation of GNNs improves and provides a scalable memory complexity, it comes at the cost of increased computation time. With mini-batches of size *b*, the memory requirement for each mini-batch of a GNN model is in *O*(*Kb* + *bp*^*K*^). The time complexity of each mini-batch is in *O*(*Kbp*^*K*^), and for *n*/*b* batches, the total time complexity of the model becomes *O*(*Knp*^*K*^).

The exponential increase in computational time to *O*(*Knp*^*K*^) from *O*(*KNp*) of that of full-batch models arises from the need to aggregate a neighborhood size of *O*(*bp*^*K*^) for each of *n*/*b* batches independently. This complexity can arise despite having a small number of nodes considered in each batch. To aggregate *K*-hop information for *b* nodes, *p*^*K*^ neighbors are to be considered; thus, a mini-batch will have to store and process *bp*^*k*^ nodes in the worst case. In our TensorFlow (TF) implementation of models reported later in the results section, we use TF Queues to handle neighbor sampling in parallel for batches to avoid stalling GPU time for this pre-processing step.

**Neighbor Sampling:** The mini-batching of GNN training and inference is memory efficient only when *bp*^*K*^ < < *n* and when it fits in memory. In many small-world cases, where the graphs are highly connected (such as a PPI, Reddit, and Blog), the neighborhood set of a small *K* may already be the whole network, making the task computationally expensive and often infeasible with respect to memory. To make this tractable, GS considers sampling *q* neighbors at every hop and, thus, aggregates information only from this *K*.*q* partial neighborhood information that is within *K*-hops from *b* nodes in a mini-batch. The resulting complexities for time are in *O*(*KnqK*) and for space in *O*(*Kb* + *bqK*).

**Figure 2:**
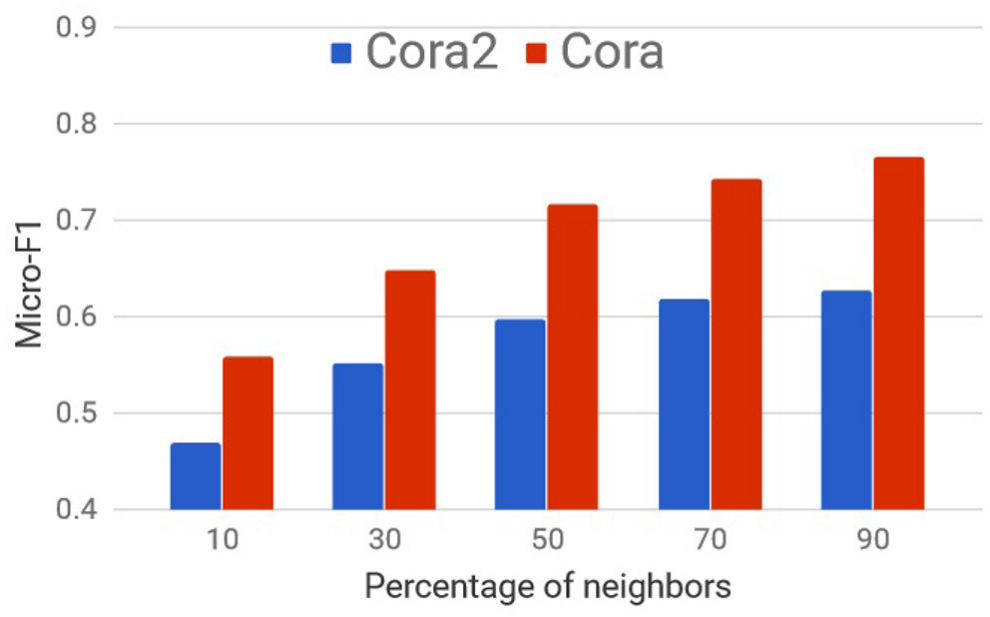
Node Information Preserving (NIP)-Mean's performance as a percentage of neighbors.

Though sampling neighbors randomly and processing information in mini-batches as with GS is scalable and fast, in many cases, it can significantly hurt the performance as shown on citation networks in Figure 2.

**Iterative GNNs:** *The mini-batch implementation of the proposed I-HOP model reduces the complexity from exponential to linear in* *O*(*TCnp*^*C*^) of the total number of hops considered with *T* iterations of a constant *C* hop differentiable kernel (*T* × *C* = *K*). Thus, I-hop has a time complexity in *O*(*Knp*^*C*^) and space complexity in *O*(*kb* + *bp*^*C*^). Run time comparison of the I-HOP model with a non-iterative GNN model is provided later in Section 8.6.

While a mini-batch version of I-HOP with neighbor sampling would further improve the computational efficiency, we do not recommend it at the cost of accuracy. Our experiments found that even *C* as small as 2 and *T* = 5 was sufficient to outperform existing methods on most of the datasets. The best models were the ones whose *C* was the largest hop which gave the best performance for the differentiable kernel. For more details on how to set *C, T*, and *K* refer to the Section 7.4.

## 6. Related Work

### 6.1. WL-Based Graph Methods and Analysis

Many extensions of classical methods have been proposed to capture higher-order relational properties of the data. *Glocalized kernels* (Morris et al., 2017) are a variant of the *k*-dimensional WL (Weisfeiler and Lehman, 1968) kernel for graph level tasks that use a stochastic approximation to aggregate information from distant nodes. The differentiable kernels are all 1-dim WL-Kernels whose direct adaptation suffers from NIM. The relation classifier (Macskassy and Provost, 2003) builds upon the homophily assumption in the graph structure and diffuses the available label data to predict the labels of unlabelled ones. To make this process more efficient, propagation kernels (Neumann et al., 2016) provide additional schemes for diffusing the available information across the graph. However, none of these provide a mechanism to adapt to the dataset by learning the aggregation filter.

Xu et al. (2018) and Li et al. (2018) presented another perspective for a specific instance of the NIM problem, which reinforces our claim. Note that their arguments are only valid for GCN's choice of F(A), Laplacian. Our approach of binomial expansions considering the α and β importance is more general and extends the results to any general aggregation kernel, F(A), considered. Numerous more recent models such as GAT (Veličković et al., 2017) can be interpreted as GCNs with F(A) determined by a self-attention mechanism. Note that, Xu et al. (2018) focus on understanding how a *K*th hop neighbor affects the *K*th hop representation of a node (we refer to this as neighborhood information morphing). However, we empirically find that understanding how the 0th hop features of a node affect its *K*th hop representation (NIM) is even more critical. Note: retaining the previous layer information of a node is not the same as preserving the original 0-hop features.

Message Passing Neural Network (MPNN) (Gilmer et al., 2017) is a message passing framework that contains the message and read-out component. They are defined for graph level tasks. Since the read-out component is specifically applicable to the graph level tasks, the left-over message component was too simple to aid us in the analysis. Hence, we proposed the I-HOP's generic propagation kernel which is more detailed than MPNN's message component, and it can additionally support iterative learning and inference. I-HOP is explicitly defined for node level tasks and aims at scaling existing graph networks.

### 6.2. Iterative Refinement Frameworks

From a dynamical systems perspective, predictive state representations (Sun et al., 2016) also make use of iterative refinement of internal representations of the model for sequential modeling tasks. However, no extension to graph models has been mentioned. In computer vision applications, iterative Markov random fields (Yu and Clausi, 2005; Subbanna et al., 2014) have also been shown to be useful for incrementally using the local structure for capturing global statistics. In this study, we restrict our focus to addressing the limitations of the current state-of-the-art differentiable graph kernels to provide higher order information for CC tasks. Moreover, I-HOP additionally leverages label information that is found to give up to a 7% point improvement on certain datasets over methods that do not use label information, refer to results for Yeast and Reddit datasets in Table 4.

Recent study, Fey et al. (2021) proposed GNNAutoscale, a framework to scale graph-based neural networks using historical embeddings similar to our I-HOP framework. GNNAutoscale pre-processes the graph and partitions the datasets into different partitions and samples batches from each partition. In I-HOP, all *C*-hop neighbors of a node are available in a batch, and historical embeddings are only used to provide neighbors' information beyond *C* hops. In contrast, with GNNAutoscale, even immediate neighbors of nodes can be missing as they can belong to another partition. Thus, they use historical embedding of out-of-batch neighbors to propagate information to nodes in the batch. GNNAutoscale can be viewed as an instance of the I-HOP framework, i.e., ${\text{Ψ}}_{k}=\Theta^{k-1}$ with $\Theta^{k-1}=h_{k-1},\forall k\in \left[1:T*C\right]$ in Equation (9) for all out-of-batch immediate neighbors.

While this results in a constant GPU memory usage similar to I-HOP, the CPU RAM usage is *K* = T*C folds more than I-HOP as the output for all the layers needs to be saved for all the nodes. Furthermore, the computational and classification performance of GNNAutoscale is highly dependent on the initial partitioning as with more inter-partition edges, the computation time will increase, and the quality of the historical embedding's approximation will depend on the homophilous nature of the graphs as even the immediate neighbors might be missing. In I-HOP, unlike GNNAutoscale, we can theoretically quantify the time complexity directly, as discussed in the previous section, which leads to an apparent exponential reduction in complexity. Our model can be less error-prone as we have the actual information up to *C* hops. Additionally, the model can better capture different label distributions in the neighborhood using label-based historical approximations. Also, unlike GNNAutoscale, where the performance can drop compared to the original GNN method even for shallow networks, we can control at what iteration (*t*) to stop with I-HOP. As a result, the performance will either saturate or increase after *C*-hops. Thus, I-HOP is more generic, flexible, CPU RAM efficient over GNNAutoscale and can leverage useful label information.

## 7. Experiments

### 7.1. Dataset Details

In this study, we treat networks as undirected graphs but the proposed framework can also handle directed graphs with non-negative edges. We extensively evaluate the proposed models and the baselines on 11 datasets from various domains. To the best of our knowledge, there exists no previous study in collective classification that reports results on these many datasets over a wider range of domains. Dataset statistics are summarized in Table 2. Code and pre-processed datasets are available at https://github.com/PriyeshV/HOPF.

**Table 2:** Dataset stats: |*V*|, |*E*|, |*F*|, |*L*|, L_*m*_ denote the number of nodes, edges, features, multi-label dataset.

**Dataset**	**Network**	**|*V*|**	**|*E*|**	**|*F*|**	**|*L*|**	**L_***m***_**
Cora (Lu and Getoor, 2003)	Citation	2708	5429	1433	7	F
Citeseer (Bhattacharya and Getoor, 2007)	Citation	3312	4715	3703	6	F
Cora2 (Mccallum, 2000)	Citation	11881	34648	9568	79	T
Pubmed (Namata et al., 2012)	Citation	19717	44327	500	3	F
Yeast (Cheng et al., 2002)	Biology	1240	1674	831	13	T
Human (Hamilton et al., 2017)	Biology	56944	1612348	50	121	T
Reddit (Hamilton et al., 2017)	Social	232965	5376619	602	41	T
Blog (Wang et al., 2010)	Social	69814	2810844	5413	46	T
Fb (Pfeiffer III et al., 2015)	Social	6302	73374	2	2	F
Amazon (Leskovec and Sosič, 2016)	Product	16553	76981	30	2	F
Movie (Cantador et al., 2011)	Movie	7155	388404	5297	20	T

Most datasets used in this study are taken directly from already available preprocessed dataset repositories. In other cases where only raw data is available, the work study follows the preprocessing instructions provided in the original studies. The train/test/Val split for 5-folds for all works is available in the above link. Additional specification to preprocess any data that is important is mentioned below along with their descriptions.

**Social Networks:** We use Facebook (FB) (Pfeiffer III et al., 2015; Moore and Neville, 2017), BlogCatalog (BLOG) (Wang et al., 2010), and Reddit dataset (Hamilton et al., 2017). In the FB dataset, the nodes are FB users and the task is to predict the political views of a user given the gender and religious view of the user as features. In the BLOG dataset, the nodes are users of a social blog directory, the user's blog tags are treated as node features and edges corresponding to friendship or fan following. The task here is to predict the interests of users and labels with less than 500 samples are removed. In Reddit, the nodes are the Reddit posts, the features are the averaged glove embeddings of text content in the post, and edges are created between posts if the same users comment on both. The task here is to predict the sub-Reddit community to which the post belongs. Unlike the original inductive task on the Reddit dataset, here, we use it for a transductive task.

**Citation Networks:** We use four citation graphs: Cora (Lu and Getoor, 2003), Citeseer (Bhattacharya and Getoor, 2007), Pubmed (Namata et al., 2012), and Cora-2 (Mccallum, 2000). In all the four datasets, the articles are the nodes and the edges denote citations. The bag-of-word representation of the article is used as node attributes. The task is to predict the research area of the article. Apart from Cora-2, which is a multi-label classification dataset from Mccallum (2000), others are multi-class datasets.

**Biological Network:** We use two protein-protein interaction (PPI) networks: Yeast and Human. The yeast dataset is part of the KDD cup 2001 challenge (Cheng et al., 2002) which contains interactions between proteins. The task is to predict the function of these genes. Additionally, we use the available gene-location information. Similarly, the Human dataset, introduced in Hamilton et al. (2017), is a PPI network from human tissues. The dataset contains PPI from 24 human tissues and the task is to predict the gene's functional ontology. Features consist of positional gene sets, motif gene sets, and immunology signatures.

**Movie Network:** We constructed a movie network from the Movielens-2k dataset available as a part of the HetRec 2011 workshop (Cantador et al., 2011). The dataset is an extension of the MovieLens10M dataset with additional movie tags. The nodes are the movies and edges are created between movies if they share a common actor or director. The movie tags form the movie features and movies with no tags are removed. The task here is to predict all possible genres of the movies.

**Product Network:** We constructed an Amazon DVD co-purchase network which is a subset of Amazon_0505 co-purchase data by Leskovec and Sosič (2016). The network construction procedure is similar to the one created in Moore and Neville (2017). The nodes correspond to DVDs and edges are constructed if two DVDs are co-purchased. The DVD genres are treated as DVD features. The task here is to predict whether a DVD will have Amazon sales rank ≤ 7,500 or not.

### 7.2. Experiment Setup

The experiments follow a semi-supervised setting with only 10% labeled data. We consider 20% of nodes in the graph as test nodes and randomly create 5 sets of training data by sampling 10% of the nodes from the remaining graph. Furthermore, 20% of these training nodes are used as the validation set. We do not use the validation set for (re)training.

### 7.3. Weighted Cross Entropy Loss

Models in earlier studies (Yang et al., 2016; Kipf and Welling, 2017), were trained with a balanced labeled set, i.e., equal number of samples for each label is provided for training. Such assumptions on the availability of training samples and similar label distribution at the test time are unrealistic in most scenarios. To test the robustness of CC models in a more realistic set-up, we consider training datasets created by drawing random subsets of nodes from the full ground truth data. It is highly likely that randomly drawn training samples will suffer from severe class imbalance. This imbalance in class distribution can make the weight updates skewed toward the dominant labels during training. To overcome this problem, we generalize the weighted cross entropy defined in Moore and Neville (2017) to incorporate both multi-class and multi-label setting. We use this as the loss function for all the methods including baselines. The weight ω for the label *i* is given in the equation below, where |*L*| is the total number of labels, and $N_{j}$ represents the number of labeled samples with label *j*. Thus, the weights for labels at training, validation, and test phases are the same. The weight of each label ω_*i*_ is inversely proportional to the number of samples having that label.


(11) $$\begin{array}{l} \omega_{i}=\frac{\sum_{j=1}^{\left|L\right|}N_{j}}{|L|\times N_{i}} \end{array}$$

### 7.4. Hyper-Parameters

The hyper-parameters for the models are the number of layers of neural network (hops), dimensions of the layers, dropouts for all layers, and L2 regularization. We train all the models for a maximum of 2,000 epochs using Adam (Kingma and Ba, 2015) with the initial learning rate set to 1e-2. We use a variant of the patience method with learning rate annealing for early stopping of the model. Specifically, we train the model for a minimum of 50 epochs and start with patience of 30 epochs and drop the learning rate and patience by half when the patience runs out (i.e., when the validation loss does not reduce within the patience window). We stop the training when the model consecutively loses patience for 2 turns. Important hyper-parameter details are tabulated in Table 3.

**Table 3:** Hyperparameters for different datasets.

**Hyperparams**	**CORA**	**CITE**	**CORA2**	**YEAST**	**HUMAN**	**BLOG**	**FB**	**AMAZON**	**MOVIE**	**Pubmed**	**Reddit**
Learning Rate	1E-02	1E-02	1E-02	1E-02	1E-02	1E-02	1E-02	1E-02	1E-02	1E-02	1E-02
Batch Size	128	128	128	128	512	512	128	512	64	128	512
Dimensions	16	16	128	128	128	128	8	8	128	16	128
L2 weight	1E-03	1E-03	1E-06	1E-6	0	1E-06	0	0	1E-06	1E-3	0
Dropouts	0.5	0.5	0.25	0.25	0	0	0	0	0	0.5	0
WCE	Yes	Yes	Yes	Yes	No	Yes	Yes	Yes	Yes	Yes	No
Activation	ReLU	ReLU	ReLU	ReLU	ReLU	ReLU	ReLU	ReLU	ReLU	ReLU	ReLU

### 7.5. Implementation Details

We found all weighted average kernels along with the GS-Max model to share similar optimal hyper-parameters as their formulations and parameters were similar. In fact, this is in agreement with the work of GCN and GS where all their models had similar hyper-parameters. However, GS-LSTM which has more parameters and a different aggregation function required additional hyper-parameter tuning. For reported results, we searched for optimal hyper-parameter setting for a two layer GCN-S model on all datasets with the validation set. We then used the same hyper-parameters across all the other models except for GS-LSTM for which we searched separately. We report the performance of models with their ideal number of differentiable graph layers, *C* based on their performance in the validation set. The maximum number of differentiable hops beyond which performance saturated or decreased on datasets were: 3 hops for Amazon, 4 hops for Cora2 and Human, and 2 hops for the remaining datasets. For the Reddit dataset, we used partial neighbors 25 and 10 in 1st and 2nd hop which is the default GS setting as the dataset had extremely high link density.

We row-normalize the node features and use Glorot initialization for weights (Glorot and Bengio, 2010). Since the percentage of different labels in training samples can be significantly skewed (Moore and Neville, 2017), we weigh the loss for each label inversely proportional to its total fraction as in Equation (11). We added all these components to the baseline codes too and ensured that all models have the same setup in terms of the weighted cross entropy loss, the number of layers, dimensions, patience based stopping criteria, and dropouts. In fact, we observed an improvement of 25.91% for GS on their dataset. GS's LSTM model gave Out of Memory error for Blog, Movielens, and Cora2 as the initial feature size was large, and with a large number of parameters for the LSTM model, the parameter size exploded. Hence, for these datasets alone, we reduced the size of the feature.

**Setting the Values C, K, and T:** The choice of K (required number of hops or message passing rounds) and the decision to use iterative learning depends on a variety of factors such as memory availability and relevance of the labels. The choice of C (the number of end-end differentiable layers) is determined by performance on the validation set. C should be set as the minimum of maximum differentiable layers that fit into memory or the maximum hop beyond which performance of validation set saturates or drops. Post finding the optimal value for *C*, the search for optimal value for *T* (number of steps of iterative inference) is motivated by the importance of information from higher hops and/or by label correlations. One solution is to set *T* to an arbitrary value, preferably determined based on the validation set. In our experiments, we searched for the optimal value of *T* corresponding to a fixed *C* for every run in the range [1, 5].

### 7.6. Models Compared

We compare the proposed NIP and I-HOP models with various differentiable WL kernels, semi-supervised ICA, and two baselines, BL_NODE and BL_NEIGH as defined in Table 1. I-NIP is an instance of HOPF which iteratively uses the NIP-MEAN kernel. BL_NODE is a K-layer feedforward network that only considers the node's information ignoring the relational information whereas BL_NEIGH ignores the node's information and considers the neighbors' information. BL_NEIGH is a powerful baseline that we introduce. It is helpful to understand the usefulness of relational information in datasets. In cases where BL_NEIGH performs poorer than BL_NODE, the dataset has less or no useful relational information to extract with the available labeled data and vice versa. In such datasets, we observe no significant gain in considering beyond one or two hops. All the models in Tables 4, 5 except SS-ICA, GCN, and GS models have skip connections. GS models combine node and neighborhood information by concatenation instead of summation.

**Table 4:** Results in micro-F1 for transductive experiments.

	**Datasets**											**Aggregate measures**	
**MODELS**	**Blog**	**FB**	**Movie**	**Cora**	**Citeseer**	**Cora2**	**Pubmed**	**Yeast**	**Human**	**Reddit**	**Amazon**	**Shortfall**	**Rank**
BL_NODE	37.929	**64.683**	50.329	59.852	65.196	40.583	83.682	59.681	41.111	57.118	64.121	12.11	8.82
BL_NEIGH	19.746	51.413	35.601	77.43	70.181	63.862	83.16	53.522	60.939	59.699	66.236	10.52	8.46
GCN	34.068	50.397	39.059	76.969	**72.991**	**63.956**	85.722	62.565	58.298	75.667	61.777	6.91	6.64
GCN-S	39.101	63.682	51.194	**77.523**	71.903	63.152	**86.432**	60.34	62.057	77.637	**73.746**	2.79	4.36
GCN-MEAN	38.541	62.651	51.143	76.081	**72.357**	62.842	85.792	61.787	64.662	74.324	63.674	3.97	6
GS-MEAN	**39.433**	64.127	50.557	76.821	70.967	62.8	84.23	59.771	63.753	79.051	68.266	3.43	6
GS-MAX	**40.275**	64.571	50.569	73.272	71.39	53.476	85.087	62.727	65.068	78.203	70.302	3.87	4.73
GS-LSTM	37.744	**64.619**	41.261	65.73	63.788	38.617	82.577	58.353	64.231	63.169	68.024	9.94	8.46
**NIP-MEAN**	**39.433**	64.286	51.316	76.932	71.148	63.901	**86.203**	61.583	**68.688**	77.262	69.136	**2.51**	**3.9**
SS-ICA	38.517	64.349	**52.433**	75.342	68.973	63.098	84.798	**68.444**	43.629	**81.92**	65.789	4.56	5.73
**I-NIP-MEAN**	39.398	62.889	**51.864**	**78.854**	71.541	**66.23**	85.341	**69.917**	**68.652**	**81.64**	**75.045**	**0.54**	**2.82**

*The lower shortfall is better. Top two results for each dataset in bold*.

**Table 5:** Results in Micro-F1 for inductive learning on human tissues.

	**Node**	**Neighbor**	**GCN-S**	**GCN-MEAN**	**GS-Mean**	**GS-Max**	**GS-LSTM**	**NIP-MEAN**	**SS-ICA**	**I-NIP-MEAN**
PPI	44.51	83.891	88.585	86.049	88.585	79.634	78.054	92.243	61.51	92.477

## 8. Results and Discussions

In this section, we make some observations from the results of our experiments as summarized in Tables 4, 5. In Table 4, we report the averaged test results for transductive experiments obtained from models trained on the 5 different training sets. We also report results on the Transfer (Inductive) learning task introduced in Hamilton et al. (2017) under their same setting, where the task is to classify proteins in new human tissues (graphs) which are unseen during training.

### 8.1. Statistical Significance

In order to report the statistical significance of models' performance across different datasets, we resort to Friedman's test and Wilcoxon signed rank test as discussed in Demšar (2006). Levering Friedmans' test, we can reject the null hypothesis that all the models perform similarly with *p* < 0.05. The statistical significance of our proposed models is provided in Section 8.5.1.

### 8.2. Model Consistency

These rank based significance tests do not provide a metric to measure the robustness of a model across datasets. One popular approach is to use count based statistics like average rank and number of wins. The average rank of the models across datasets is provided in the table, where a lower rank indicates better performance. It is evident from Table 4 that the proposed algorithm I-NIP-MEAN achieves the best rank and wins on 4/11 datasets followed by SS-ICA with 2 wins and NIP-Mean with 1 win and second best rank. By this simple measure of rank and number of wins, the proposed method outperforms other models overall.

However, we argue that this is not helpful in measuring the robustness of models. For example, there could be an algorithm that is consistently the second best algorithm on all the datasets with minute difference from the best and yet have zero wins. To capture this notion of consistency, we introduce a measure, *shortfall*, which captures the relative penalty in performance compared to the best performing model on a given dataset.


(12) $$\begin{array}{l} shortfall\left[model\right]=\underset{D_{i}\in \left\{\text{datasets}\right\}}{\sum }\frac{best\left[D_{i}\right]-model\left[D_{i}\right]}{\left|\text{datasets}\right|} \end{array}$$

Where *best* $\left[D_{i}\right]$ is the micro_f1 of the best performing model and *model* $\left[D_{i}\right]$ is the model's performance, for the dataset $D_{i}$. The total number of datasets is denoted by |datasets|. *shortfall* of models are reported in Table 4.

Even using this consistency measure the proposed algorithm I-NIP-MEAN outperforms existing methods. In particular, notice that while SS-ICA seemed to be the second best algorithm using the naive method of counting the number of wins, it does very poor when we consider the *shortfall* metric. This is because SS-ICA is not consistent across datasets and, in particular, it gives a very poor performance on some datasets which is undesirable. On the other hand, I-NIP-MEAN not only wins on 4/11 datasets but also does consistently well on all the datasets and, hence, has the lowest *shortfall* and also the best average rank.

### 8.3. Baselines vs. CC Models

As mentioned earlier, the baselines BL_NEIGH and BL_NODE use *only neighbor* and *only node* information, respectively. *In datasets, where BL_NEIGH significantly outperforms BL_NODE, all CC models outperform both these baselines by jointly utilizing the node and neighborhood information*. In datasets such as Cora, Citeseer, Cora2, Pubmed, and Human, where the performance of BL_NEIGH > BL_NODE, CC models improve over BL_NEIGH by up to 8% in the transductive setup. Similarly, on the inductive task where the performance of BL_NEIGH is greater than BL_NODE by ≈ 40%, CC methods end up further improving by another 8%. In Reddit and Amazon datasets, where the performance of BL_NODE ≈ BL_NEIGH, CC Methods still learn to exploit useful correlations between them to obtain a further improvement of ≈ 20% and ≈ 10%, respectively.

### 8.4. WL-Kernels vs NIP-Kernels

**NIM in WL-kernels:** The poor performance of BL_NEIGH compared to BL_NODE on the Blog, FB, and Movie datasets suggest that the neighborhood information is noisy and node features are more crucial. *The original GCN which aggregates information from the neighbors but does not use CONCAT or skip connections typically suffers a severe drop in performance of up to* ≈ 13% *on datasets with a high degree*. Despite having the node information, GCN performs worse than BL_NODE on these datasets. The improved performance of GCN over BL_NEIGH in Blog and Movie supports the claim that node information is essential.

**Solving NIM With Skip Connections:** The original GCN architecture does not allow for skip connections from *h*_0_ to *h*_1_ and from *h*_*K*−1_ to *h*_*K*_. We modify the original architecture and introduce these skip connections (GCN-S) by extracting *h*_0_ features from the 1st convolution's node information. *With skip connections, GCN-S outperforms the base GCN on 8/11 datasets*. We observed a performance boost of ≈ 5 − 13% in Blog, FB, Movie, and Amazon datasets even when we consider only 2 hops, thereby decreasing the *shortfall* on these datasets. GCN-S closed the performance gap with BL_NODE on these datasets and in the case of the Amazon dataset, it further improved by another 9%. GCN-MEAN which also has skip connections performs quite similarly to GCN-S in all datasets and does not suffer from NIM as much as GCN. *It is important to note that skip connections are required not only for going deeper but more importantly, to avoid information morphing even for smaller hops*. GS models do not suffer from the NIM issue as they concatenate node and neighborhood information. Authors of GS also noted that they observed a significant performance boost with the inclusion of the CONCAT combination. GS-MEAN's counterpart among the summation models is the GCN-MEAN model which gives a similar performance on most datasets, except for Reddit and Amazon where GS-MEAN with concat performs better than GCN-MEAN by ≈ 5%. GS-MAX provides very similar performances to GS-MEAN, GCN-MEAN, and GCN-S across the board. Their shortfall is also very similar. The poor performance of GS-LSTM might be because of the morphing of earlier neighbors' information by more recent neighbors in the list.

**Solving NIM With NIP Kernels:** *NIP-MEAN, a MEAN pooling kernel from the NIP propagation family outperforms its WL family counterpart, GCN-MEAN on 9/11 datasets. With Wilcoxon signed-rank test, NIP-MEAN* > *GCN-MEAN with p* < *0.01*. It achieves a significant improvement of ≈ 3 − − 6% over GCN-MEAN in Human, Reddit, and Amazon datasets. It also outperforms GS-MEAN on another 9/11 dataset despite GS-MEAN having two times more parameters. NIP-MEAN provides the most consistent performance among the non-iterative models with a shortfall as low as 2.51. NIP-MEAN's clear improvement over its WL-counterparts demonstrates the benefit of using the NIP family of kernels which explicitly mitigates the NIM issue.

### 8.5. Iterative Inference Models vs. Differentiable Kernels

Iterative inference models, SS-ICA and I-NIP-MEAN exploit label information from the neighborhood and scale beyond the memory limits of differentiable kernels. This was evidently visible with our experiments on the large Reddit dataset. Reddit was computationally time-consuming with even partial neighbors due to its high link density. However, the iterative models scale beyond 2 hops and consider 5 hops and 10 hops for SS-ICA and I-NIP-MEAN, respectively. This is computationally possible because of the linear scaling of time and the constant memory complexity of iterative models. Hence, they achieve superior performance with lesser computation time on Reddit. The micro-f1 scores of SS-ICA over *T* = 1 − 5 iterations on a particular fold for Reddit dataset was 56.6, 78.4, 79.8, 81.9, 82.2, and 82.2. Similarly for I-NIP-MEAN on the same fold, we obtained 78, 80.1, 80.7, 81, 81.4, and 81.7. SS-ICA's performance of 81.92 starting from 57.118 (*BL*_*NODE*) as seen in the table shows that iterative models are remarkable despite not being end-end differentiable.

The benefit of label information over attributes can be analyzed with SS-ICA which aggregates only the label information of immediate neighbors. In the Yeast dataset, SS-ICA gains ≈ 8 − − 10% improvement over non-iterative models which do not use label information. However, SS-ICA does achieve good performance on some datasets as it does not leverage neighbors' features and is restricted to only learning first-order local information differentiably.

#### 8.5.1. Iterative Differentiable Kernels vs. Rest

I-NIP-MEAN which is an extension of NIP-MEAN with iterative learning and inference can leverage attribute information and exploit non-linear correlations between the labels and attributes from different hops. *I-NIP-MEAN improves over NIP-MEAN on seven of the eleven datasets with a significant boost in performance up to* ≈ 3 − 8% *in Cora2, Reddit, Amazon, and Yeast datasets*. Levering Wilcoxon signed-rank test, I-NIP-MEAN is significantly better than NIP-MEAN (with p < 0.05). I-NIP-MEAN also successfully leverages label information like SS-ICA and obtains a similar performance boost on Yeast and Reddit datasets. *It also outperforms SS-ICA on eight of eleven datasets with a statistical significance of p* < *0.02 as per the Wilcoxon test*. The benefits of using neighbors' attributes along with labels are visible in Amazon and Human datasets where the I-NIP-MEAN model achieves ≈ 10% and ≈ 25% improvement correspondingly over SS-ICA which uses label information alone. *Moreover, by leveraging both attributes and labels in a differentiable manner, it further achieves a* 3% *improvement over the second best model in cora2. This superior hybrid model, I-NIP-MEAN emerges as the most robust model across all datasets with the lowest shortfall of* 0.54.

#### 8.5.2. Inductive Learning on Human Dataset

For the inductive learning task (Table 5), the CC models obtain a 44% improvement over BL_NODE by leveraging relational information. The I-NIP-MEAN and NIP-MEAN kernels achieve the best performance with a ≈ 6% improvement over GCN-MEAN.

### 8.6. Run Time Analysis

**Figure 3:**
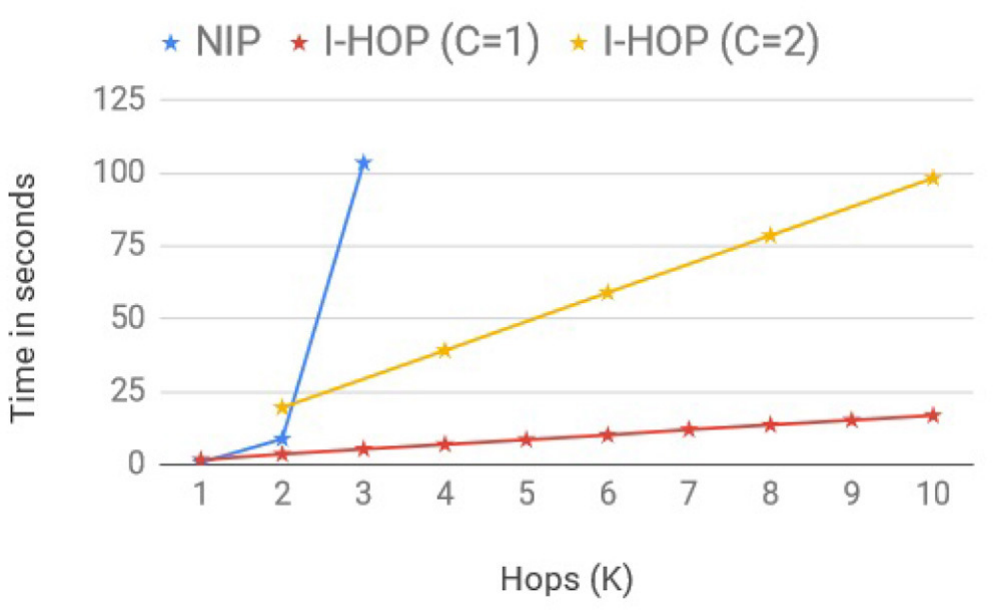
Linear scaling of iterative models.

We adapt the scalability setup from GCN (Kipf and Welling, 2017) to compare the average training time per epoch between the fully differentiable model, NIP-MEAN and iterative differentiable model, I-NIP-MEAN, to make predictions with multi-hop representations. We consider two I-HOP-Variants here: I-NIP-MEAN with 1 differential layer (C = 1) and I-NIP-MEAN with 2 differential layers (C = 2). In order to obtain multi-hop representations, we increase the number of iterations, *T* accordingly. Note: I-NIP-MEAN with C = 2 can only provide multi-hop representations of multiples of 2. The training time, included time to pre-fetch neighbors with queues, forward pass of NN, loss computation, and backward gradient propagation similar to the setup of Kipf and Welling (2017). We record wall-clock running time for these models to process a synthetic graph with 100k nodes, 500k edges, 100 features, and 10 labels on a 4GB GPU. The batch size and hidden layers size were set to 128. The plot of the averaged run time overruns across different hops is presented in Figure 3.

The fully differentiable model, NIP-MEAN incurred an exponential increase in compute time with an increase in the hop, (*C* = *K*) and moreover ran out-of-memory after 3 hops. Whereas, I-NIP-MEAN with *C* = 1 and *C* = 2 has a linear growth in computing time with increasing *T*. This is in agreement with the time complexity provided earlier for these models. Not only does the time for non-iterative methods increase exponentially with hops, but the memory complexity also increases exponentially with a new layer as it is required to store the gradients and activations for all the new neighbors introduced with a hop. In comparison, the runtime of the proposed iterative solution has a linear growth rate and also has a lesser memory footprint.

## 9. Conclusion

In this study, we proposed I-HOP, a novel framework for CC that combines differentiable graph kernels with an iterative stage. Deep learning models for relational learning tasks can now leverage I-HOP to use complete information from larger neighborhoods without succumbing to over-parameterization and memory constraints. For future study, we can further optimize the framework by committing only high confidence labels, like in cautious ICA (McDowell et al., 2007) to reduce the erroneous information propagation, and we can also increase the supervised information flow to unlabeled nodes by incorporating ghost edges (Gallagher et al., 2008). The framework can also be extended for unsupervised tasks by incorporating structural regularization with Laplacian smoothing on the embedding space.

## Data Availability Statement

The original contributions presented in the study are included in the article/supplementary material, further inquiries can be directed to the corresponding author/s.

## Author Contributions

PV: developed the framework and analysis. PV and YC: developed the model and performed the experiments. BR, MK, and SP: helped PV analyze and present the experiment results. PV: wrote the manuscript with support from YC and guidance from BR, MK, and SP. BR: supervised the project through its entirety.

## Funding

This study was partly supported by a grant from Intel Technology India Pvt., Ltd (No. RB1819CSE002INTIBRAV) to BR and MK. The funder was not involved in the study design, collection, analysis, interpretation of data, the writing of this article or the decision to submit it for publication.

## Conflict of Interest

The authors declare that the research was conducted in the absence of any commercial or financial relationships that could be construed as a potential conflict of interest.

## Publisher's Note

All claims expressed in this article are solely those of the authors and do not necessarily represent those of their affiliated organizations, or those of the publisher, the editors and the reviewers. Any product that may be evaluated in this article, or claim that may be made by its manufacturer, is not guaranteed or endorsed by the publisher.
